# Lexical category acquisition is facilitated by uncertainty in distributional co-occurrences

**DOI:** 10.1371/journal.pone.0209449

**Published:** 2018-12-28

**Authors:** Giovanni Cassani, Robert Grimm, Walter Daelemans, Steven Gillis

**Affiliations:** Center for Computational Linguistics and Psycholinguistics (CLiPS), University of Antwerp, Antwerp, Belgium; Universidad Catolica de Valencia San Vicente Martir, SPAIN

## Abstract

This paper analyzes distributional properties that facilitate the categorization of words into lexical categories. First, word-context co-occurrence counts were collected using corpora of transcribed English child-directed speech. Then, an unsupervised *k*-nearest neighbor algorithm was used to categorize words into lexical categories. The categorization outcome was regressed over three main distributional predictors computed for each word, including frequency, contextual diversity, and average conditional probability given all the co-occurring contexts. Results show that both contextual diversity and frequency have a positive effect while the average conditional probability has a negative effect. This indicates that words are easier to categorize in the face of uncertainty: categorization works best for words which are frequent, diverse, and hard to predict given the co-occurring contexts. This shows how, in order for the learner to see an opportunity to form a category, there needs to be a certain degree of uncertainty in the co-occurrence pattern.

## 1 Introduction

Lexical category acquisition defines the process by which children start to productively use words according to categorical restrictions, e.g. not using a noun where a verb is required or using a noun where it is needed. Lexical category acquisition consists of using words in appropriate new contexts, not only in contexts where they were already encountered [1–3]. In order to explain this process, much attention has been devoted to the analysis of what in the input children get during their first years can bootstrap the process of lexical category acquisition [4]. Among several proposals, distributional bootstrapping [5] suggests that children can exploit the co-occurrence patterns across linguistic units, such as words or morphemes. The underlying idea can be traced back to the observation that a word can be known “by the company it keeps” [6], meaning that the syntactic and semantic behavior of a target word can be determined by looking at which other words it co-occurs with in the language. Therefore, according to the distributional bootstrapping hypothesis, children can learn lexical categories by tracking co-occurrence patterns: nouns will share similar contexts, that are different from the contexts that verbs share [7].

Many computational simulations and corpus studies [8–23] as well as behavioral studies [24–32] have corroborated the distributional bootstrapping hypothesis. Moreover, this learning strategy has proven effective for several languages other than English, including French [33], Dutch [34], Spanish [35], German [36, 37], Turkish [37], Chinese [38], and several other typologically diverse languages [39].

The main goal of studies on distributional bootstrapping has been that of assessing how accurately words can be categorized given certain distributional contexts, in order to identify the type of distributional information children may capitalize on to learn that words behave categorically. Researchers have addressed questions such as (i) words of which lexical category are categorized better [13, 14] or (ii) which model or which type of distributional information yields better categorization [18, 22]. However, no study systematically addressed item-level factors in lexical category acquisition. It is argued here that it is necessary to investigate which properties of the input give rise to the observed developmental patterns in the context of lexical category acquisition. The current study aims to start filling in this gap by analyzing which distributional properties of words make them easier to categorize into lexical categories using distributional co-occurrences. The next sub-section describes three types of information that distributional co-occurrences provide and which may play a role in determining which words are easier to categorize.

### 1.1 The information in distributional co-occurrences

#### 1.1.1 Frequency

Given the scarcity of studies that addressed individual factors in word categorization, it is not straightforward to decide which distributional predictors to consider in order to model categorization accuracy. One candidate is frequency, which is known to have a positive effect on word learning, with more frequent words being learned sooner and better [40, 41]. It can thus be hypothesized that it also has a positive influence on word categorization: if a word occurs more often, the occasions for inferring its categorical restrictions increase. However, it is also true that function words are only grouped at later developmental stages [20] while being the most frequent words in a child’s learning experience. In line with this potentially ambiguous effect of frequency, a study by Roy et al [42] showed that frequency had a strong facilitatory effect on the production of nouns (more frequent nouns were produced earlier) but almost irrelevant for closed-class words. This hints to the possibility that frequency is not the only factor playing a role in word learning, and possibly also in word categorization.

#### 1.1.2 Contextual diversity

A second candidate predictor is diversity, which is at the center of several recent studies in the context of language acquisition. A study by Vergara-Martinez and colleagues [43] showed that the neural correlates of diversity differ from the neural correlates of frequency, showing similar characteristics to the effect of semantic richness. This suggests that frequency and diversity capture different aspects of language processing and should be kept distinct. Diversity can be broadly defined as the degree of variability of the contexts in which a linguistic item occurs. Its effect has been investigated on several linguistic processes, including lexical latencies for real words [44] and for non-words in an artificial language learning task [45], word learning [46–48], spontaneous verb production [49], and vocabulary growth, non-word repetition, and sentence recall [50]. Using both computational modeling and behavioral experiments, it was shown that diversity had a positive effect on learning and production, often a more important effect than frequency.

However, diversity has been defined differently across studies: as the number of different movies in which a target word occurs in a subtitle corpus [43]; as the number of types of text in which a target word occurs [48]; as the number of different documents and passages in which a target word occurs [44]; as the number of different co-occurring words in a window around the target word [46]; as the number of different possible referents with which a target word label occurs in a cross-situational learning study [47]; as the number of different sentence-picture combinations in which a target word is found in the context of an artificial language learning task [45]; as the number of types in a corpus [50]. The approach taken in this study is closer in spirit to the study by Hills et al [46]: diversity is defined as the number of different distributional contexts with which a word co-occurs and is referred to as contextual diversity. It is a much more local measure of diversity than those which considered movies or document types [43, 44, 48] and is purely linguistic, unlike measures which considered referents as well [45, 47].

The main reason for focusing on local distributional contexts to define diversity is that this study aims to assess which factors affect categorization *when using co-occurrence counts with local distributional contexts* to learn lexical categories. It follows that measuring contextual diversity as the number of different contexts with which a word co-occurs is the most straightforward approach. Moreover, in a previous work of ours [51], it was shown that more local distributional contexts provide more information to learn lexical categories, hence the decision to focus on local distributional contexts. Furthermore, considering diversity at the level of other units would be problematic. For example, measuring diversity as the number of utterances in which a word occurs could collapse different usages which provide variable distributional contexts, which may in turn carry different information to infer the lexical category of the word. Other measures like number of different chunks, syntactic constituents, or similar units with a more transparent syntactic nature would introduce a further degree of freedom in the modeling strategy: different results (and biases) could arise depending on which specific algorithm or coding strategy would be used.

Beyond its role in facilitating several processes in language acquisition and processing, our previous work explicitly linked words’ contextual diversity to lexical category acquisition [51]. In that work, corpus-based computational simulations were carried out to analyze which distributional predictors influence how useful a context is to the learning of lexical categories. The usefulness of distributional contexts was quantified using methods from machine learning and regressed over several distributional predictors, including context frequency, lexical diversity (i.e. the number of different words a distributional context co-occurs with), and predictability (i.e. how well can the occurrence of a distributional context be predicted given that a certain word has been observed). It was shown that the predictability of a context given the co-occurring words has a negative effect on context usefulness, once frequency and diversity are controlled for. This means that distributional contexts tend to be more useful to learn lexical categories when they are harder to predict given the co-occurring words. To better understand this effect, consider a toy example with two contexts, *the_X* and *you_X*: both occur a hundred times and both co-occur with three different words, *the_X* co-occurs with *dog, cat, cow* while *you_X* co-occurs with *run, cry, eat*. Predictability of contexts given words depends on word frequencies and word co-occurrences with all contexts in the corpus: therefore, let us assume that *dog* occurs 40 times, 30 times with *the_X*—and therefore 10 times with other contexts; *cat* occurs 50 times, 45 of which with *the_X*; *cow* occurs 25 times, always with *the_X*. Therefore, the average conditional probability of *the_X* given the co-occurring words is (30/40 + 45/50 + 25/25)/3 = (0.75 + 0.9 + 1)/3 = 0.88. On the contrary, *run* occurs 150 times, 30 with *you_X*; *cry* occurs 100 times, 25 times with *you_X*; and *eat* occurs 200 times, 45 with *you_X*. Therefore, the average conditional probability of *you_X* given co-occurring words is (30/150 + 25/100 + 45/200)/3 = (0.2 + 0.25 + 0.225)/3 = 0.225. Evidence reported in [51] suggests that the latter context is more useful to infer the lexical category of the co-occurring words. From this observation and assuming that words which are easier to categorize co-occur with useful contexts, the prediction is derived that higher contextual diversity facilitates word categorization. If a context is hard to predict given co-occurring words, it means that these words co-occur with other contexts to a large degree. It can be the case that each word co-occurs very often with only one other context, having low diversity and thus contradicting the prediction. However, on average, it is expected to be more likely that words which do not predict a certain context well co-occur with many different contexts rather than with only one, hence the prediction that contextually diverse words should be categorized better.

#### 1.1.3 Predictability

A third distributional predictor that has been at the center of several studies on language acquisition is predictability, which has been typically operationalized using forward or backward transitional probabilities [52–54] and studied in the context of word segmentation. It was shown that children are sensitive to this piece of distributional evidence and can rely on it to segment the speech input. Predictability was also investigated in relation to segmentation of larger chunks and multi-word units, with reported effects on language acquisition and processing [55–58]. Moreover, two previous studies connected predictability to lexical category acquisition and are therefore relevant to the current analysis. First, our aforementioned study [51] reported a positive effect of lexical diversity on context usefulness, indicating that contexts tend to be more useful to infer the lexical category of co-occurring words when they co-occur with many different words. From the observation that contexts tend to be more useful when they are lexically diverse, it is hypothesized that words which are easier to categorize should be on average harder to predict given co-occurring contexts. If a useful context co-occurs with many words, it should be harder to correctly predict which of the many candidate words will co-occur with the context. This hypothesis dovetails with evidence reported by Matthews and Bannard [59]. In their study, evidence was provided that children find it easier to group words together when these co-occur with diverse distributional contexts, controlling for context frequency. Therefore, the degree to which words are predictable given the co-occurring distributional contexts appears to be a relevant predictor in lexical category acquisition which should be investigated further. Consider again two contexts, e.g. *my_X* and *look_at_X*. In this toy example, both occur a thousand times, but the first co-occurs with a hundred words, the second co-occurs with only five words. Previous studies [51, 59] showed that the first is more useful to learn about the lexical category of the co-occurring words. If it is assumed that words co-occurring with more useful contexts are easier to categorize, then words co-occurring with the context *my_X* should be easier to categorize than those co-occurring with *look_at_X*. Moving from distributional properties of contexts to those of words, each word co-occurring with *my_X* is harder to predict given the context (there are 99 competitors, although each with a different degree of predictability given by its co-occurrence frequency with the target context). These words, however, all co-occur with (a variable number of) other contexts: the prediction is that if, across all the contexts they co-occur with, these words are on average hard to predict given the contexts themselves, then they should be easier to categorize.

The fact that two predictions about the potential effect of distributional properties on word categorization are derived from the analysis of what makes contexts useful shows the tight relation between them. In our previous work, it has been shown that useful contexts tend to be diverse and unpredictable [51]. On the basis of this finding, it is predicted that words should be categorized better when they too are diverse and unpredictable, provided that the assumption that words can be categorized better when they co-occur with useful contexts holds. If a context is diverse, co-occurring words should have low predictability. If a context is unpredictable, co-occurring words should have high diversity. If our previous results about context usefulness and the hypotheses about word categorization derived from them are borne out, word categorization would come out as a process which is facilitated by uncertainty in distributional patterns. When words always and only appear in the same distributional contexts, it would be impossible to learn higher-order clusters which depend on distributional context, since there would be no evidence that other words can occur in that context or that one of the target words can also occur elsewhere. Learning such clusters, however, becomes possible, and is facilitated, when variability is introduced in co-occurrence patterns. This puts categorization at odds with prediction: a language in which learners can always easily predict upcoming items would be a language in which it is hard to categorize the items, and vice versa. At a very speculative level, it seems likely that languages are optimized for both tasks, trading off linguistic cues which make prediction easier with other cues which facilitate categorization.

The main goal of this study is thus to bring attention to the necessity of assessing which factors determine whether a word is categorized correctly or not based on distributional information, to help constrain models of categorization by highlighting the pieces of information they should be sensitive to. Three predictors are going to be evaluated: frequency, contextual diversity, and predictability, as previously described.

### 1.2 How can words be categorized?

In order to evaluate the effect that the aforementioned predictors have on categorization accuracy, a model of word categorization is required, whose outcome can be taken as the target variable to be modeled. The literature on distributional bootstrapping offers three main ways in which categories have been conceived and categorization effectiveness evaluated. The biggest difference is between categorization approaches which posit the existence of categories during learning and models which do not. The first approach can be further divided into models which assign words to categories and models which assign categories to words.

An example of the models which do not assume the existence of categories while learning comes from the seminal study by Redington et al [21]. Words were represented as vectors of co-occurrences over high frequency context words occurring in the vicinity of the target words. Word vectors were then clustered using hierarchical clustering. Categorization effectiveness was evaluated by cutting the hierarchy of categories at different levels and computing accuracy and completeness of each cluster. The former reflects the proportion of words in a cluster which share their lexical category out of all the words in the cluster. On the contrary, the latter indicates the proportion of words in a cluster which share their lexical categories out of all the words in the input which were tagged with that category. Gold-standard lexical categories do not influence learning, but are only used in the evaluation: changing the set of Part-of-Speech (PoS) tags would not change the dendrogram, but will only affect the categorization measure. Moreover, categories do not need to be granted psychological existence for the model to work: the only necessary device is a measure to compute similarity. Categories are an epiphenomenon of the hierarchy, not the base of it: they only appear when the hierarchy is cut for evaluation purposes. This approach to categorization is consistent with the theoretical positions expressed by Ambridge [60], and Ramscar and Port [61] that a behavior which can be described categorically does not necessarily depend on an explicit categorization process that attaches labels to items.

In a similar vein, the approach proposed by Mintz [16] and known as the *frequent frames* approach does not need correct lexical categories to be available in order to learn. However, unlike the model proposed by Redington et al. [21], it assumes that each context actually *is* a category. In this account of distributional bootstrapping, words are categorized together when they co-occur with the same context: it does not matter how often they co-occur with it and which other contexts they co-occur with, the moment two words co-occur with the same context they are taken to belong to the same category. Therefore, words and categories enter a many-to-many relations. In this account, distributional contexts act as category labels that are attached to words. Categorization effectiveness was evaluated as in the model by Redington et al. [21], by computing accuracy and completeness for each context (remember that a context is also a category), and then averaging over contexts to obtain a summary score. Again, changing the set of target PoS tags would not change the category structure but simply the categorization score.

A third approach to categorization, in which categories are assumed to have psychological reality and are assigned to words, is exemplified by the *flexible frames* model proposed by St. Clair et al [22]. Here, a feed-forward neural network was trained to predict the PoS tag of a word based on the co-occurring context. While previous approaches could be applied to both tokens and types, this is an eminently token-based approach: for each token, distributional information is used to predict a category label. Therefore, different tokens of the same type can be categorized differently according to their context of occurrence. Moreover, here PoS tags are attached to words: each word belongs to a category, and is explicitly marked. Learning that words behave according to categories is operationalized by assigning the right category to each word. The most important aspect of this learning model, however, is that unlike both previous accounts, learning depends on the set of target categories: changing it would not only change the categorization score, but it would alter the representations on which categorization depends. This happens because the neural network actively tries to predict the correct category for each word based on the context of occurrence, and updates its weights to minimize errors. Changing the set of PoS tags would change the error signal and therefore the network’s representations.

All these accounts, however, present shortcomings that will be addressed in the approach to categorization implemented in the current work. The hierarchical clustering proposed by Redington et al. [21] models categorization as a hierarchical problem, with categorization effectiveness changing according to the level in the dendrogram which is inspected. *Frequent frames*, on the contrary, have the drawback that categories heavily depend on which contexts are considered: changing the type of context may yield comparable categorization effectiveness, but the category structure would change dramatically because different categories would be used. This is undesirable because such a system is rigid to change. Finally, categorization displayed by the *flexible frames* model is dependent on the set of PoS tags: different categories may again yield similar categorization scores, but coming from a completely different representation.

In order to tackle these problems, the proposed algorithm to categorize words will have the following features. In line with the approach by Redington et al. [21], and agreeing with the theoretical positions on the possibility of finding categorically constrained behavior in the absence of categories as independent constructs [60, 61], the model does not assume the existence of categories to model categorization: words are clustered based on their similarity, computed over their vectors of co-occurrences, without being labeled with a category or assigned to a category. Moreover, the set of PoS tags used to evaluate categorization will not influence learning, in line with the models by Redington et al [21] and Mintz [16]. Finally, unlike the model by Redington et al. [21], the proposed model will not attempt to learn a hierarchical structure but categorize words based on local similarity alone. Summing up, the categorization algorithm used in this study will be similarity-based, local, flat, and unsupervised. Whether such an algorithm can actually provide an effective categorization is an empirical question that will also be elucidated in this work. This algorithm has the advantage of providing a unique categorization outcome for each word: this categorization outcome can then be regressed over the predictors of interest, i.e. word frequency, its contextual diversity, and its average predictability given co-occurring contexts. Crucially, categorization (i) will not depend on the level in the hierarchy (as would happen in the model by Redington et al. [21]); (ii) will not depend on a specific context (as would happen in the *frequent frames* model [16]) with words potentially being classified correctly in one context and incorrectly in another; and (iii) will not depend on the set of target PoS tags (as would happen in the *flexible frames* model [22]). This model (which will be described in detail in following sections) thus offers a convenient way to address the research question which motivated this study, which concerns the properties that make words easier to categorize using distributional information.

### 1.3 Research question

Summing up, the current study aims to answer the following research question: which distributional properties of words in the linguistic input children receive make words easier to categorize into lexical categories on the basis of their distributional patterns of co-occurrence? Three distributional factors are considered: frequency, contextual diversity and predictability given co-occurring contexts. Finally, a similarity-based, local, flat, and unsupervised algorithm for lexical category acquisition is used, in order to obtain a binary categorization outcome to be used as the dependent variable in all subsequent analyses.

## 2 Materials and methods

### 2.1 Corpora and pre-processing

In order to carry out the categorization experiment, two transcribed corpora of English child-directed speech were downloaded from the CHILDES database [62]: the Manchester corpus [63] and the Suppes corpus [64]. The first consists of data collected from 12 children, recorded between 21 and 36 months of age. The second corpus consists of data from one child, Nina, recorded between the ages of 24 and 40 months. Thus, the analyses reported in this paper make use of 13 individual longitudinal sub-corpora.

For each individual sub-corpus, the following pre-processing steps were applied. The utterances from all the adult speakers were isolated, preserving the chronological order in which they were uttered. The PoS tag of each token was retrieved from the MOR tier of the CHILDES annotation scheme and associated with it. No lemmatization was performed. Following evidence from [12] indicating that utterance boundaries provide useful contextual information, the beginning and end of each utterance were considered as relevant distributional contexts, indicated by two dummy symbols added to every utterance. Finally, each PoS tag from the CHILDES tag-set was mapped to one of 5 coarser tags: nouns (including pronouns), verbs (including auxiliaries and non-finite forms), adjectives, adverbs, and function words, collapsing all other categories. This coarser tag-set was used to evaluate categorization and is chosen in order to focus on content words.

Each corpus was then processed chronologically, one utterance at a time. For each lexical element in an utterance (thus excluding utterance boundaries), two bigrams and three trigrams were considered. For example, given the utterance

#start *the* *dog* *barked* *at* *the* *angry* *postman* #end

and the target word *dog*, the following lexically specific distributional contexts were considered: *the*_X, X_*barked*, #start_*the*_X, X_*barked*_*at*, and *the*_X_*barked*—X marks the empty slot where target words are located. These contexts were chosen to be rather local and to include the lexically specific contexts investigated in previous studies on distributional bootstrapping [16, 18, 22, 51].

Homographs which were found in the corpora tagged with more than one PoS tag were kept distinct, so that, given the utterances *Lead is heavy* and *They lead us*, the words *is* and *us* are considered to be co-occurring with two different contexts, *lead-1_X* and *lead-2_X*, even though both follow the string *lead*. At the same time, different co-occurrence counts were collected for *lead-1* and *lead-2*, so that the first co-occurs with the context X_*is* while the second co-occurs with the context X_*us*. This strategy was adopted for all homographs regardless of whether they are also homophones or not, so the verb *open* was also differentiated from the adjective *open*. This strategy was adopted for two reasons. First, the transcription norms arbitrarily determine whether two homophones are also homographs: the verb *can* and the noun *can* are both homophones and homographs, while the preposition *to*, the adverb *too*, and the numeral *two* are all homophones but not homographs. Since the goal of this study is to model language acquisition in children who only access speech, relying on the transcription to decide what counts as the same context and what does not would be biased by conventional transcription norms which do not apply to the case at hand. Second, differentiating all contexts gives us the possibility to fully analyze the input and its properties: this study aims to characterize the amount and kind of information children can access, not the information they do access. However, once the question of what information is available has been answered, it can be tested whether children’s behavior is consistent with what was predicted given the analysis of the information in the input.

While traversing each corpus, a word-context co-occurrence matrix was updated every time a target word co-occurred with a context. No restrictions were applied: all words and all contexts were considered to build the co-occurrence matrix. Each corpus was processed from the first to the last utterance, using a longitudinal approach. First, word-context co-occurrences were collected on the first 40% of the utterances in a corpus, and a categorization experiment was performed. Then, word-context co-occurrences were collected on the first 50% of the utterances in a corpus, and a new categorization experiment was performed. This procedure was repeated on the first 60%, then 70%, up to 100% of the utterances in a corpus. This strategy ensures that the time course of categorization can be evaluated, by looking at how categorization accuracy for each word evolves over time. The decision to start at 40% was taken to ensure that distributional statistics were computed on a substantial portion of each input corpus even at the first time point while still having enough sufficiently spaced time points.

### 2.2 Learning algorithm

In order to perform the categorization experiment, an unsupervised *k*-nearest neighbor (*k*NN) approach was chosen [65, 66]. The fact that the method is unsupervised entails that the correct PoS tags are not used during learning, although data annotated with PoS information are used for evaluation purposes. Every word is reduced to its co-occurrence vector over all the possible distributional contexts. At every time point and for all words encountered thus far, the distance between the target word and all other words in the co-occurrence matrix is computed using a leave-one-out approach, to ensure that the target word cannot be retrieved as its own nearest neighbor. It is important to underline that the learning algorithm is still unsupervised even though it makes use of annotated data: correct PoS tags are not used to train the algorithm or to improve learning, but only to evaluate the model’s performance. Once the distance from the target has been computed for all words in the co-occurrence matrix, words are sorted according to how close they are to the target. Finally, the closest one is selected as the nearest neighbor. It is of course possible that more than one word is found at the closest distance: in this case, the most frequent neighbor out of all the nearest neighbors is chosen. If two or more nearest neighbors also have the same frequency, then one is chosen at random among the most frequent ones, since the distributional information cannot discriminate between them. Consider the situation in which the word *squirrel* is being categorized. The *k*NN algorithm retrieves ten neighbors, all at the same distance from the co-occurrence vector of *squirrel*. Six of the neighbors have frequency 1 in the corpus, one neighbor has frequency 5, and three neighbors have frequency 10: *mouse, tree, and brown*. Since more than one nearest neighbor was retrieved, the frequency heuristic is used, which keeps the three most frequent neighbors. Since they all have the same frequency, one is selected at random: two are nouns, one is an adjective. Therefore there are two out of three possibilities to sample a noun (which would be the correct choice). When more than one nearest neighbor is retrieved, the higher the proportion of words from a same category in the pool of neighbors, the higher the chance that a word from that category is selected, and vice versa. In the long run, if distributional information can discriminate words from different lexical categories, the situations where the frequency heuristic and the random sampling are going to be used will decrease, and even when they are used, the chances of retrieving a word which has the correct lexical category will increase.

Since nearest neighbors are retrieved based on their distance from the target, the choice of which distance metric is used is critical. Among the many options, cosine was selected as it proved robust in distributional learning over many tasks and implementation choices [67]. Cosine similarity is computed according to Eq 1, where **A** and **B** are two non-zero vectors of length *n*.

$$\begin{array}{c} cosine\left(A,B\right)=1-\frac{A\cdot B}{∥A∥∥B∥}=1-\frac{\sum_{i=1}^{n}A_{i}B_{i}}{\sqrt{\sum_{i=1}^{n}A_{i}^{2}}\sqrt{\sum_{i=1}^{n}B_{i}^{2}}} \end{array}$$ (1)

Cosine distance is computed by dividing the product of the two vectors by the product of their vector norms, and then subtracting this value from 1, which is the highest possible value for the cosine between two vectors. Therefore, lower values indicate more similar vectors. Cosine is relative insensitive to absolute frequency values, since it measures the size of the angle between two vectors. Consider two 3-dimensional vectors **A** = [1, 0, 9] and **B** = [0, 10, 90]. Their cosine distance is 0.012, hence very small, even though the element-wise differences are not all small. The point is that both vectors have a high value in the last position (high relative to the other values in each vector). If **A** and **B** are taken to represent words and each position indicates a context of occurrence, they would have very different overall frequency, but similar co-occurrence patterns, since they both tend to co-occur with the third context. Cosine captures this similarity and is especially driven by the last value in both vectors, in spite of a large absolute difference.

The other important parameter in the proposed categorization algorithm is the number of neighbors to consider, indicated by the parameter *k*, which was set to 1. Other values could be chosen, expanding the pool of neighbors from which to sample. However, the goal of this study is not that of finding the best instantiation of a *k*NN model for lexical category acquisition. The goal is to uncover which distributional properties of the words being categorized make categorization easier or harder. The choice to keep the model as local as possible by considering only the neighbor(s) at the closest distance from the target makes sure that the effect of the predictors of interest are interpreted in the context of a local categorization approach.

Categorization was evaluated by checking whether the PoS tag of the target word matches the PoS tag of its nearest neighbor: as it was highlighted before, this does not mean that the learning algorithm was trained using a supervised approach. Learning happens without knowing the PoS tag related to a word context vector, avoiding the use of supervision. Furthermore, unlike other *k*NN approaches where tie-breaking makes use of the correct PoS tags to check whether the majority of neighboring words belongs to a certain category, the approach used in this study makes sure that categories are entirely left out of the learning process. In other words, the categorization algorithm does not have access to the information that the word *squirrel* is a noun: it has only access to the co-occurrence vector associated to *squirrel* and retrieves other co-occurrence vectors which maximize the similarity to the target, again without knowing or being influenced by the lexical category of the words associated to each vector. In order to investigate which distributional factors influence categorization, item-level categorization accuracy as evaluated by comparing the PoS tag of the target word and its nearest neighbor was considered as the binary dependent variable: if the PoS tags matched, the word was considered a *hit*, while in case of a mismatch the word was considered a *miss*. The categorization outcome was taken as a proxy of how easy it is to categorize a word under the proposed approach: *easy* words will be classified correctly using distributional information more often than *hard* words.

### 2.3 Independent variables

In order to evaluate how distributional learning changes over time, a categorization experiment was performed at seven moments for each individual sub-corpus. Therefore, time is a predictor of interest: it was coded as an ordinal variable taking values between 0 and 6, mapping to 40% and 100% of the input corpus respectively. Time was operationalized as the percentage of corpus processed, to model the effect of increasing exposure to the input language on how words are grouped into lexical categories. It is hypothesized that time has a positive effect on categorization effectiveness, since larger portions of the input language should provide more robust and informative distributional co-occurrences. Moreover, the following distributional properties were computed for each word and used as independent variables to model categorization accuracy.

*Frequency*, i.e. how often a word occurs in the input corpus. Frequency counts were logged, following evidence showing that lexical processing is better modeled using log-frequencies [68–70]. This transformation is also in line with the Weber-Fechner law from psychophysics [71] stating that people do not perceive two numerically equal differences in the same way when numbers grow larger. Word frequency is predicted to have a positive effect on word categorization, making frequent words easier to categorize. This hypothesis is motivated by the observation that frequency has a beneficial effect in language acquisition across the board [40]. However, on the basis of frequency alone, words such as *the* or *of* would be expected to be categorized very easily, and more easily than words such as *kitten* or *eat*, contrary to psycholinguistic and developmental evidence [2, 20, 72, 73].

*Contextual diversity*, i.e. how many different distributional contexts a word co-occurs with (distributional contexts being lexically specific bigrams and trigrams, used to update the word-context co-occurrence matrix as previously described). Contextual diversity was also logged, following the reasoning with frequency above. It is hypothesized to have a positive effect on categorization accuracy based on the prediction formulated in [51], where it was reported that contexts which are on average harder to predict given the co-occurring words are more useful to learn lexical categories. If the assumption is made that *easy* words co-occur with useful contexts, then *easy* words should show a higher contextual diversity. However, the same caveat about function words mentioned while discussing the predicted effect of frequency applies here, since function words tend to co-occur with a large variety of contexts, definitely larger than nouns and verbs, whose co-occurrence patterns are more constrained.

*Average conditional probability* of words given contexts, i.e. how easy it is to predict a word given a context, averaged over all the contexts a word co-occurs with. This measure quantifies predictability. In detail, for each context which co-occurred with a word, the word-context co-occurrence count was divided by the context frequency count. The resulting conditional probabilities were finally averaged to yield a summary measure of predictability for each single word. Two previous studies can inform the prediction concerning this effect. First, Matthews and Bannard [59] provided evidence that children find it easier to cluster words that co-occur in diverse contexts (holding frequency constant). If a context co-occurs with many different words, then it is harder to predict which individual word will occur given the context. Therefore, average conditional probability should have a negative effect: the harder it is to predict which specific word will occur once a context has been observed, the easier it should be to categorize that word. The same effect was predicted by our previous results [51]: analyzing distributional predictors of contexts’ usefulness, they showed that useful contexts tend to be diverse. A low predictability signals a very variable co-occurrence pattern, where several other words could be observed co-occurring with a certain context (e.g. the words which may occur before or after a determiner). It is hypothesized that this variability in co-occurrence patterns facilitates lexical category acquisition.

*Median Information Gain* (IG) of the co-occurring contexts, taken to summarize how useful the contexts with which a word co-occurs are. IG [74, 75] is a feature weighting approach used in machine learning to increase the importance of more useful pieces of information in a categorization task. The idea is thus to find which distributional contexts are more useful to the categorization task and make sure they have a higher impact on which category is chosen. Following our previous study [51], IG is taken as a measure of how useful a context is. IG at every time point was computed independently for each context. Then all the contexts co-occurring with a word were sorted according to their IG value and the median was taken, since the median is more robust to skewed distributions than the mean. The idea is that the higher the median IG value, the higher the usefulness of the co-occurring contexts. Median IG is included to validate the assumption that *easy* words co-occur with useful contexts and it is thus predicted to have a positive effect. If it were shown to have a negative effect, the predictions that have been sketched out so far would not hold any longer, as they are all based on the idea that *easy* words co-occur with useful contexts. Thus, median IG is included as a sanity check to test the hypotheses put forward for the previous independent variables: it is not of theoretical interest but is included to corroborate or disprove previous predictions.

### 2.4 Statistics

Given that the dependent variable is binary, logistic mixed-effects models [76] were used, in order to control for corpus and word identity with the inclusion of by-corpus and by-word random intercepts and slopes. Random slopes were included to account for the auto-correlations between categorization outcomes for the same word at different time points: if a word was categorized correctly at time 4 it is likely that it will be categorized correctly at time 5 as well, and this may bias estimates if not duly controlled. The statistical analysis is carried out in *R* using the *lme4* package [77]. Visualizations are realized combining the *ggplot2* package [78] and the *effects* package [79]. Model selection is carried out by first selecting the random structure that best accounts for the data and then adding fixed effects, evaluating the improvement in fit with the Akaike Information Criterion (AIC, [80]).

Importantly, the statistical analysis was restricted to words that occur in all the thirteen sub-corpora and making sure that they occurred at least in the first 50% of each input sub-corpus, so as to have at least six measures on each continuous predictor. The first restriction is motivated by the goal of analyzing the words that are most representative of the linguistic input of English-learning children, avoiding idiosyncratic items. The second constraint is applied to counter the potential presence of too many missing values, which would make the analysis less robust. Finally, a third constraint was introduced, discarding all individual observations that have a frequency of 1 in a sub-corpus and at a given time point, to avoid perfect correlation between two predictors, as a word which only occurred once has a perfectly determined diversity value.

## 3 Study 1

The first analysis focused on evaluating each predictor of interest individually, without controlling for the others. Fitting a separate model for each predictor makes sure to avoid collinearity issues that may confound results. After having determined the best random structure consisting of by-corpus random intercepts and by-word random intercepts and slopes, each predictor was added to the baseline model, consisting of time as a fixed effect and the aforementioned random component. Time is part of the baseline model as it is required by the presence of by-word random slopes, but is not a predictor of interest in this analysis. The improvement in fit caused by each predictor over the baseline model was computed using the AIC.

### 3.1 Results

**Fig 1:**
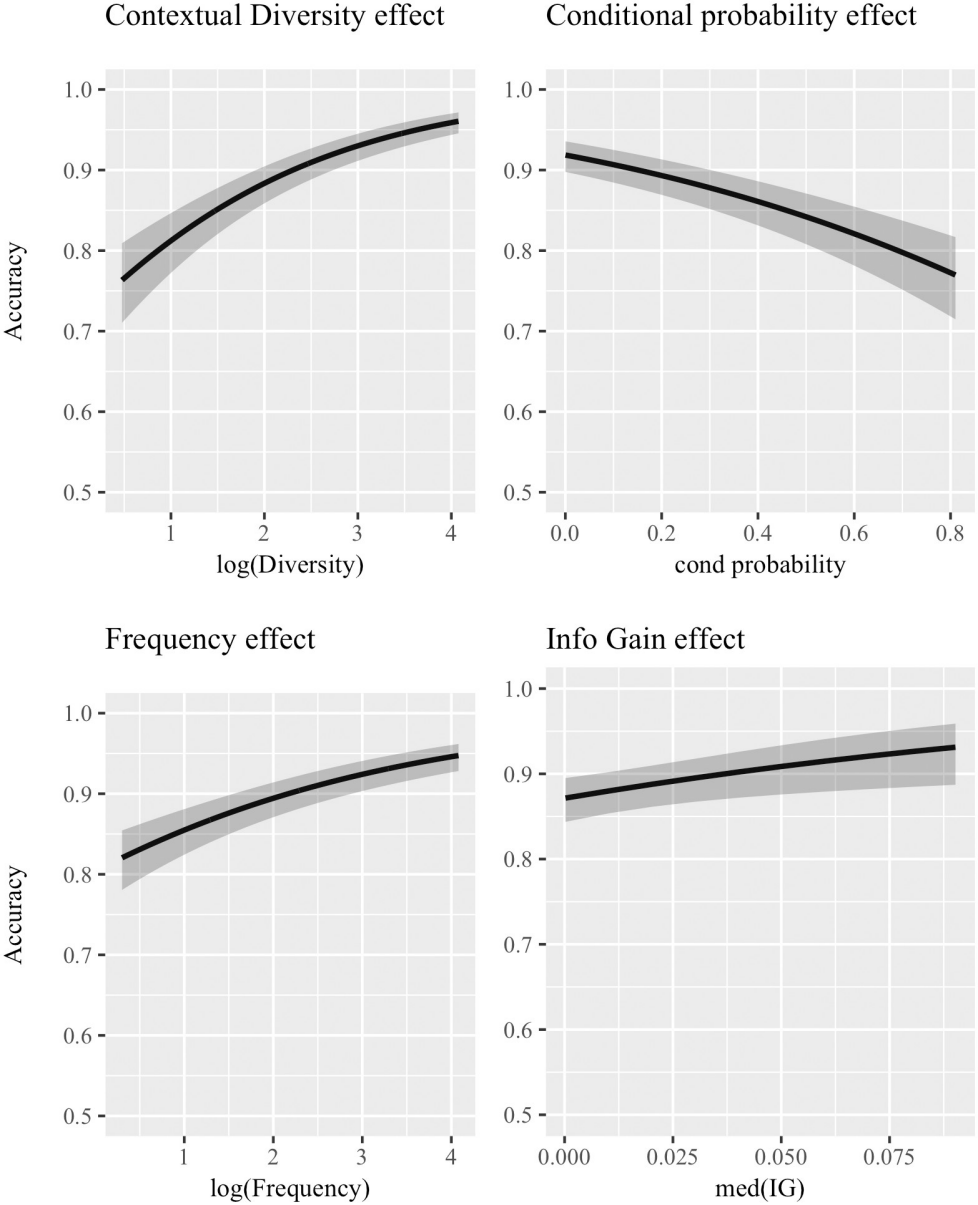
Logistic mixed-effect models for single predictors—Cosine distance. Effects of the logistic mixed-effect models which included *a single predictor* (inputted in the model together with Time, which is part of the baseline model) using categorization accuracy achieved with **cosine** as distance metric as the dependent variable. Subplots are ordered according to the reduction in AIC caused by adding the predictor to the baseline model: the most important predictor is in the top-left panel, then top-right, bottom-left, and finally bottom-right. 95% confidence bands are shown.

Table 1 shows the output of the four statistical models fitted for each predictor of interest. The predictor name is indicated in the leftmost column, followed by the *β* coefficient, the standard error (*se*) and the Z statistic. The last three columns provide indications of model’s fit: first the AIC, which is used to rank predictors from the most to the least important; then, the marginal and conditional *r*^2^ are reported (m*r*^2^ and c*r*^2^ respectively) which indicate the amount of variance explained by the fixed effects alone and by the fixed and random effects combined. Fig 1 graphically presents the effects of the four predictors.

**Table 1:** Logistic mixed-effect models for single predictors—cosine distance.

Predictor	*β*	*se*	*Z*	AIC	m*r*^2^	c*r*^2^
Baseline				35480.8	0.0045	0.6605
Contextual diversity	0.5616	0.0591	9.511 (***)	35394.5	0.0138	0.6556
Conditional probability	-1.5025	0.1831	-8.208 (***)	35416.4	0.0085	0.6605
Frequency	0.3630	0.0488	7.439 (***)	35428.8	0.0104	0.6553
Median IG	7.6771	2.9893	2.568 (*)	35476.8	0.0048	0.6608

Outcome of the logistic mixed-effect models fitted using accuracy obtained using **cosine** as the distance metric to identify nearest neighbors as the dependent variable. Each model includes *a single predictor* (together with Time, which is part of the baseline model). Significance codes:

(***): *p* < .001;

(*): *p* < .05.

Importantly, median IG has a significantly positive effect (*β* = 7.6771, *Z* = 2.518, *p* < .01), although not a strong one. The magnitude of the coefficient is misleading, since it reflects a 1 unit increase in median IG, while the range of values is very small, as shown in the bottom-right plot in Fig 1. Also, the decrease in AIC determined by median IG is the lowest, as is the m*r*^2^. Nevertheless, the significantly positive effect suggests that *easy* words do indeed co-occur with more useful contexts. Thus, the other predictors can be evaluated in the light of previous hypotheses. First, contextual diversity comes out as the most useful predictor to model categorization accuracy, with a significantly positive effect (*β* = 0.5616, *se* = 0.0591, *Z* = 9.511, *p* < .001). Second is conditional probability, with a significantly negative effect (*β* = −1.5025, *se* = 0.1831, *Z* = −8.208, *p* < .001). Frequency also has a significantly positive effect (*β* = 0.3630, *se* = 0.0488, *Z* = 7.439, *p* < .001). All reported coefficients match the predicted effects. However, the extremely low m*r*^2^ values cannot be ignored: all effects are indeed significant, but they do not explain much variance in categorization accuracy.

### 3.2 Discussion

First, the positive effect of the median IG of co-occurring contexts shows that the assumption that *easy* words, i.e. words which tend to be categorized correctly across corpora and at different time points, tend to co-occur with useful contexts is warranted, implying that the hypotheses which were formulated for each independent variable rely on a reasonable assumption. Second, conditional probability of words given contexts has a strong negative effect. This matched the prediction formulated on the basis of previous work [51, 59] which, with the use of computational simulations and behavioral experiments, showed that useful contexts tend to be lexically diverse.

Moreover, the statistical analysis revealed that both frequency and contextual diversity have a positive effect on how easy it is to categorize a word using distributional co-occurrences. This observation matches predictions formulated on the basis of results presented in our previous work [51], where it was shown that distributional contexts tend to be more useful to learn lexical categories when they are hard to predict given co-occurring words. Moreover, diversity appears to be more important than frequency in explaining categorization accuracy. This pattern matches the one reported about which factors determine how useful a distributional context is to learn lexical categories, where lexical diversity was also found to explain more variance than frequency [51]. These two pieces of evidence suggest that when it comes to learning lexical categories, diversity is a more important factor than frequency, although both effects point in the same direction. This conclusion dovetails with evidence about the strong and positive role of diversity in language learning [45–50]. Study 2 will add to the insights derived from Study 1 by considering all predictors in the same statistical model, to assess whether effects change magnitude and direction once other predictors are controlled for, or whether some predictors ceases to be significant.

## 4 Study 2

The second study evaluated whether the patterns highlighted during the first analysis hold when controlling for the other variables, in order to check whether collinearity plays a role. One important caveat has to be made: all predictors are actually collinear, especially frequency and contextual diversity. However, since every method for collinearity reduction has drawbacks [81], it was preferred not to directly address it: having provided individual analyses for each predictor, it is easier to see where and to what extent collinearity may be an issue. The baseline model is the same as in Study 1: each predictor is added to it checking whether it causes a decrease in the AIC. Predictors were added one by one until no improvement was observed over the simpler model or convergence issues arose. Time was again part of the baseline model, but in this study it is also a predictor of interest, since it is important to evaluate how categorization accuracy changes over time, once other relevant factors are taken into account.

### 4.1 Results

**Fig 2:**
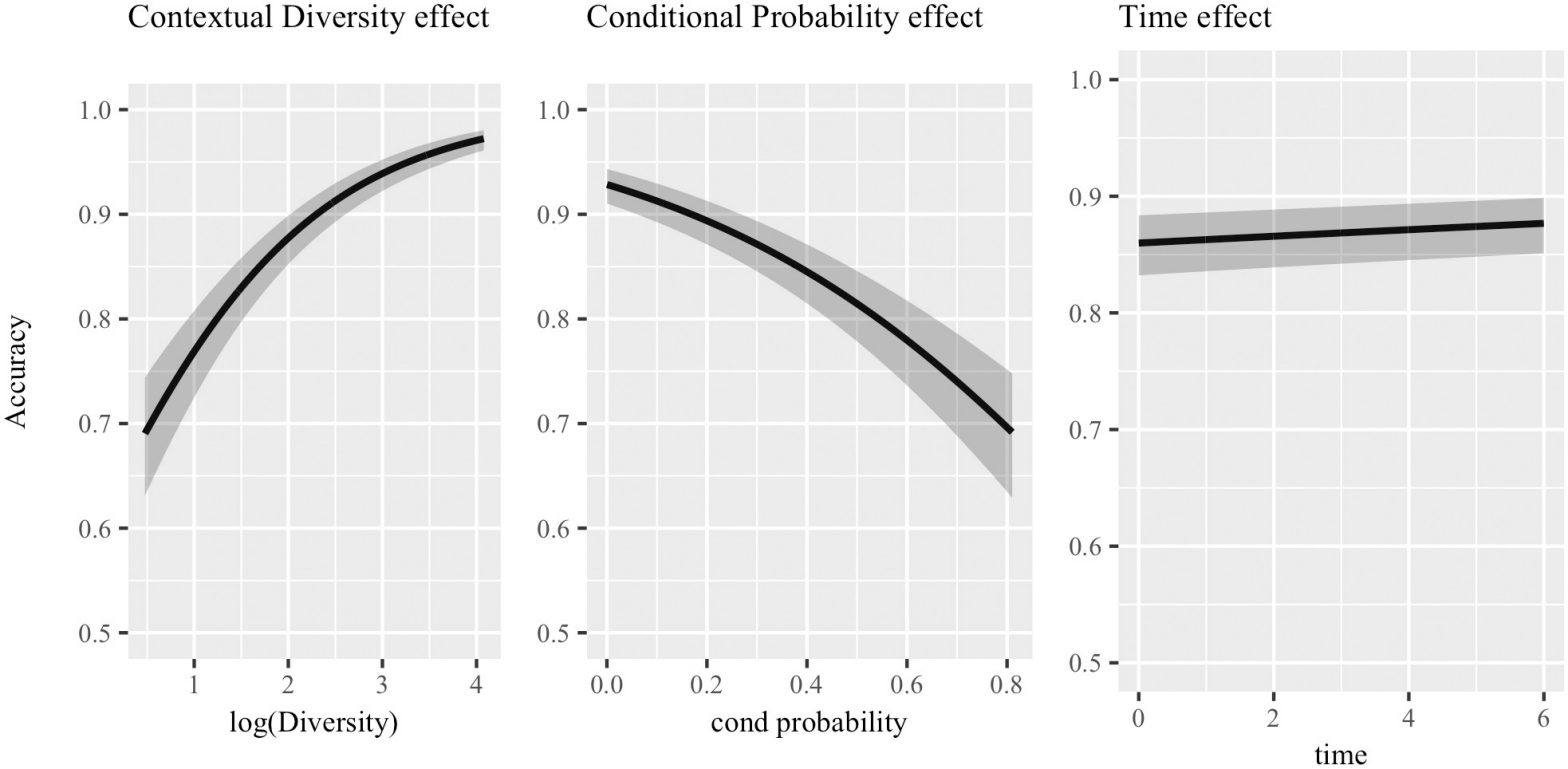
Logistic mixed-effect model including all predictors—Cosine distance. Graphical representation of the effects arising from the logistic mixed-effect mode fitted considering *all significant predictors* at once and accuracy achieves using **cosine** to retrieve nearest neighbors as the dependent variable. 95% confidence bands are shown for each effect, and predictors are ordered from left to right according to how much they improve the model fit.

Table 2 summarizes results from this statistical analysis, first providing the predictor, then its *β* coefficient and corresponding standard error (*se*), and the Z statistics. The marginal *r*^2^ of the full model is 0.0153, while the conditional *r*^2^ is 0.6475. Fig 2 shows the reported effects graphically.

**Table 2:** Logistic mixed-effect model including all predictors—Cosine distance.

Predictor	*β*	*se*	*Z*
(Intercept)	1.0363	0.1507	6.875 (***)
Time	0.0493	0.0116	4.268 (***)
Contextual diversity	0.7652	0.0622	12.310 (***)
Conditional Probability	-2.1666	0.1913	-11.324 (***)

Outcome of the logistic mixed-effect model fitted considering *all significant predictors* at once on categorization outcomes obtained using **cosine** as the distance metric to retrieve nearest neighbors. Significance codes:

(***): *p* < .001.

Contextual diversity is the most important predictor and has a positive effect (*β* = 0.7652, *se* = 0.0622, *Z* = 12.310, *p* < .001), while conditional probability comes second with a significant negative effect (*β* = −2.1666, *se* = 0.1913, *Z* = −11.324, *p* < .001). Finally, time has a significant positive effect (*β* = 0.0493, *se* = 0.0116, *Z* = 4.268, *p* < .001), confirming that being exposed to larger portions of input language allows the model to better categorize words. All other predictors did not increase the model’s fit.

**Fig 3:**
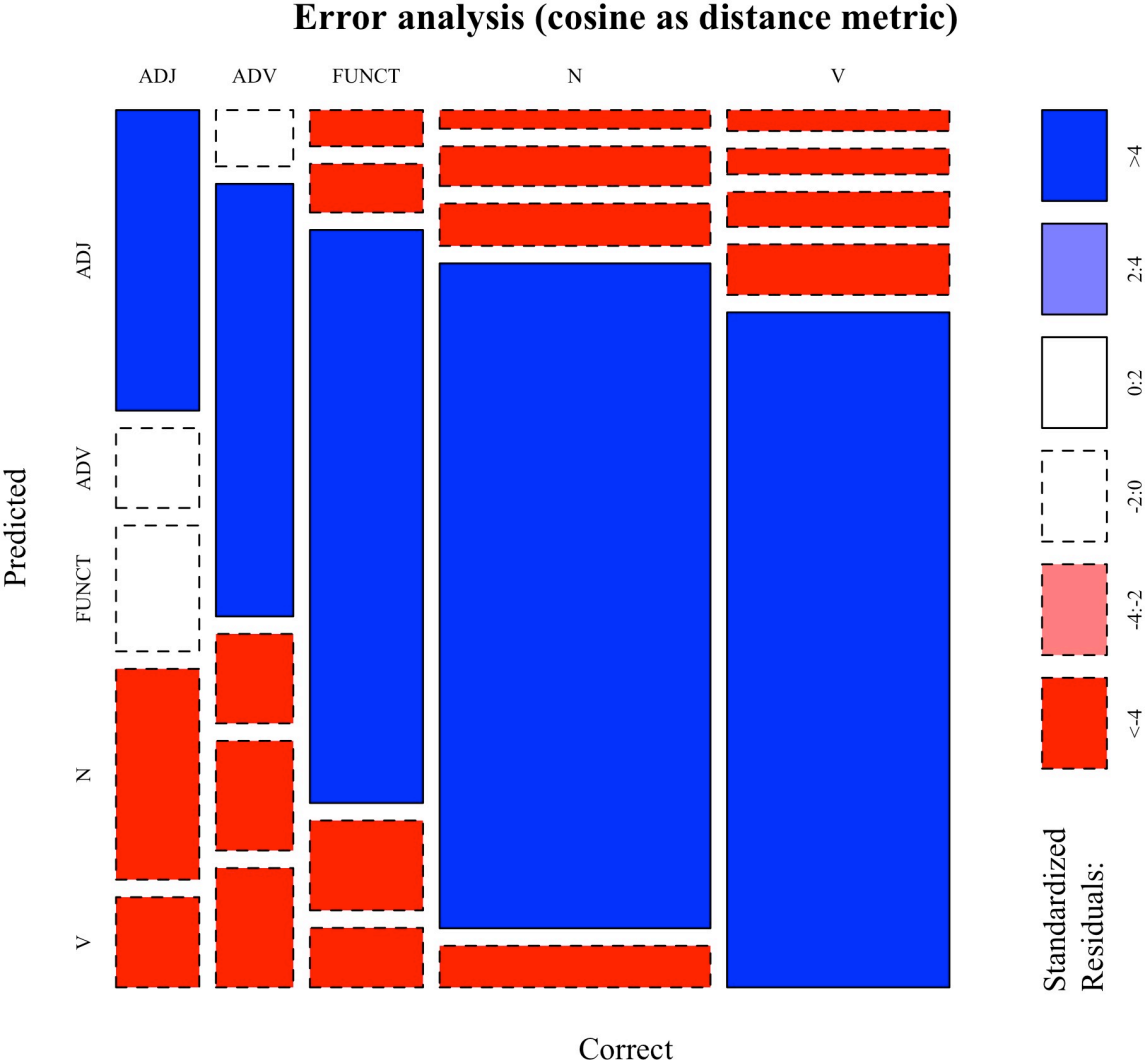
Cosine-based classification—Error analysis. Mosaic plot showing the error analysis of the categorization experiment performed using **cosine** as the distance metric to retrieve nearest neighbors. Blue cells indicate cases in which the observed frequencies significantly exceed the expected frequencies under the assumption of independence between Correct and Predicted PoS tags, while red mark the opposite scenario. ADJ: adjectives; ADV: adverbs; FUNCT: function words; N: nouns; V: verbs. For a detailed explanation of how to interpret the plot, refer to the main text.

The error analysis provided in Fig 3 using a mosaic plot shows a breakdown of categorization outcomes by PoS tag. This visualization shows how often each pair of levels of two (or more) categorical variables occurs in the data, and whether this observed count is (significantly) higher or lower than it would be expected if the variables were independent. In detail, the *x* axis in Fig 3 shows the correct categories: the width of each column indicates how many words from each category there are in the data set, so that larger columns indicate more frequent PoS tags. The *y* axis indicates the predicted categories, i.e. the categories that the *k*NN algorithm assigned to the test items: here, the height of each box indicates how often a category was predicted for each of the five correct ones. Thus, larger boxes indicate the most frequent pairings of correct and predicted PoS tag. Finally, the color (together with the line type of the box contours) indicates in which direction the observed frequencies deviate from the expected frequencies. Blue (and solid line) indicates larger observed frequencies than expected, while red (and dashed line) indicates the opposite. Darker hues indicate larger differences. White boxes indicate non-significant deviations from the expected frequencies.

After having explained how to interpret the plot, four interesting patterns can be observed. First, all the dark blue cells are on the main diagonal, indicating that the model predicts the correct category for each PoS tag more often that it would be expected by chance, i.e. by a model that did not learn anything about lexical categories. Second, the highest blue boxes are the ones for nouns and verbs, followed by function words, then adverbs and finally adjectives, indicating that categorization is particularly effective for nouns and verbs and least effective for adjectives. Third, most off-diagonal cells are red, indicating lower observed frequencies than expected. The only white cells coincide with adjectives categorized as either adverbs or function words, and with adverbs categorized as adjectives. This indicates that the model has a hard time figuring out the difference between adjectives and adverbs based on their distributional footprints. Fourth, there are many adjectives being categorized as nouns: from a distributional point of view this makes intuitive sense, as the distributional contexts in which adjectives can occur are rather similar to those of nouns, especially when considering that prenominal adjectives often occur after determiners. Summing up, this error analysis shows that categorization is most effective for nouns and verbs, while it is worse for adverbs and adjectives; finally, it also shows that errors make intuitive sense.

### 4.2 Discussion

Several interesting points are worthy of an in-depth discussion. First of all, the local, similarity-based and unsupervised approach to categorization worked well, as shown by Fig 3. This suggests that the effects shown in the regression analysis are trustworthy, given that they arise in the presence of effective categorization. At the same time, however, the remarkably low marginal *r*^2^ shows that, while significant, the distributional predictors that were considered only scratch the surface of which factors influence whether a word is classified correctly using distributional co-occurrences computed over lexically specific contexts. Given that the categorization algorithm only had access to distributional information, there must be other factors that contribute to a much larger extent to explaining why a word is categorized correctly. Interactions are likely to play a role, but they were not explored in the current study because of collinearity issues and to keep the analysis simple.

A potentially problematic result highlighted by the error analysis is to be found in the comparable categorization accuracy of nouns and verbs, which deviates from behavioral evidence reporting a consistent noun advantage in categorization [1, 72, 73]. However, it was not claimed that the current categorization algorithm describes how children implement lexical category acquisition: an ideal learner approach was purposefully taken to provide a complete analysis of what the input offers in terms of distributional learning cues. Preliminary research suggests that the noun-verb asymmetry could be motivated by the fact that children only have access to incomplete distributional information, due to their reduced learning capacities and their learning biases [13, 14]. Thus, the current results would provide an upper bound on categorization. Alternatively, this could mean that lexical category acquisition is first driven by non-distributional factors.

Delving into the results of the statistical analysis, conditional probability is again reported to have a significant negative effect, with less predictable words being categorized better. This piece of evidence appears to be the most robust, as (i) it is consistent with the analysis reported in Study 1; (ii) it is consistent with the prediction formulated based on the observation that distributional contexts are more useful when lexically diverse [51]; and (iii) it reflects behavioral evidence that children more readily categorize words which co-occur with lexically diverse contexts and cannot thus be easily predicted given the contexts themselves [59].

Moreover, it can be noted how frequency is no longer a significant predictor once contextual diversity is controlled for, suggesting that, in this simulation, diversity not only explains all the variance frequency explains, but it actually explains variance frequency does not account for. This positive effect reported for contextual diversity even after other predictors are controlled for dovetails with the prediction formulated in [51] that *easy* words should be contextually diverse given that useful contexts tend to be less predictable given the words they co-occur with.

However, it is necessary to verify that the reported effects are not due to theoretically irrelevant aspects of the design. The most important one is the distance metric: cosine was not chosen based on behavioral evidence suggesting that children compute similarity in a similar way. On the contrary, it was mainly a convenience choice, motivated by its observed robustness and widespread use in studies on the role of distributional information in language learning. Other choices are possible, which implement different strategies to compute similarity. In order to check the extent to which the effects reported for frequency, contextual diversity, and predictability generalize, Studies 1 and 2 were replicated using a different distance metric to retrieve nearest neighbors. Numeric overlap [82] was selected as a second distance metric, because it provides a different approach to similarity than cosine. Eq 2 describes how numeric overlap is computed, following the definition provided in [82]. **A** and **B** are two *n*-dimensional, non-zero vectors.

$$\begin{array}{c} numeric\_overlap\left(A,B\right)=\sum_{i=1}^{n}abs(\frac{A_{i}-B_{i}}{max(i)-min(i)}) \end{array}$$ (2)

Suppose that **A** and **B** refer to rows in a matrix, while *i* is an index over columns. First, the difference between the values at position *i* of vectors **A** and **B** is computed. Then, this difference is scaled to the interval [0; 1] by dividing it by the range of values over the corresponding column *i*. The absolute value of the scaled difference is taken. These operations are repeated for each position in the input vectors, summing the element-wise distances to yield the global distance. Element-wise distances are scaled to the same interval to contextualize absolute values. A difference of 90 between two vectors on a particular position could be very large if the maximum value across all the vectors is 100, but rather low if the maximum value is 9000. In the first case, the scaled distance for that particular position would be 0.9, while it would be 0.01 in the second (assuming that the minimum value is 0 in both cases).

Unlike cosine, numeric overlap is rather affected by differences in frequency of occurrence. Consider again the vectors used to elucidate how cosine works, i.e. **A** = [1, 0, 9] and **B** = [0, 10, 90], and assume that there is a third vector **C** = [50, 30, 2]. The numeric overlap distance between vectors **A** and **B** is 1.24. It is hard to interpret this number since there is no theoretical boundary to numeric overlap, but the important point is that the main component of this distance value is the distance on the third position of the vectors, which is 0.92. The large absolute difference, albeit scaled, affects the overall distance to a large extent. Since there is no evidence of how children compute distance functions, it was decided to investigate two very different alternatives to verify whether results observed using cosine hold or not. Replications of Studies 1 and 2 in which numeric overlap is used are detailed in Study 3.

## 5 Study 3

Study 3 aimed to further test the validity of the evidence reported in Studies 1 and 2 by changing an important aspect of the design, which however does not have any reported theoretical meaning in the context of lexical category acquisition. Since a *k*NN algorithm was used to test categorization, the choice of the distance metric used to retrieve nearest neighbors is crucial: different metrics can yield very different categorization patterns, which may be influenced differently by the predictors of interest. Therefore, in order to avoid reporting partial effects or drawing unwarranted generalizations, it was decided to consider categorization based on a different distance metric than cosine. As it is predicted that this manipulation should not reflect any theoretically meaningful aspect of development, it is predicted that the same patterns reported for cosine should be observed.

Study 3 consists of two analyses which replicate Study 1 and Study 2 respectively: the first tests the effect of each predictor of interest on categorization accuracy obtained retrieving nearest neighbors based on numeric overlap distance. The second analysis describes a single statistical model in which all relevant predictors are included at once, to evaluate how their effects change while controlling for other predictors of interest.

### 5.1 Results

**Fig 4:**
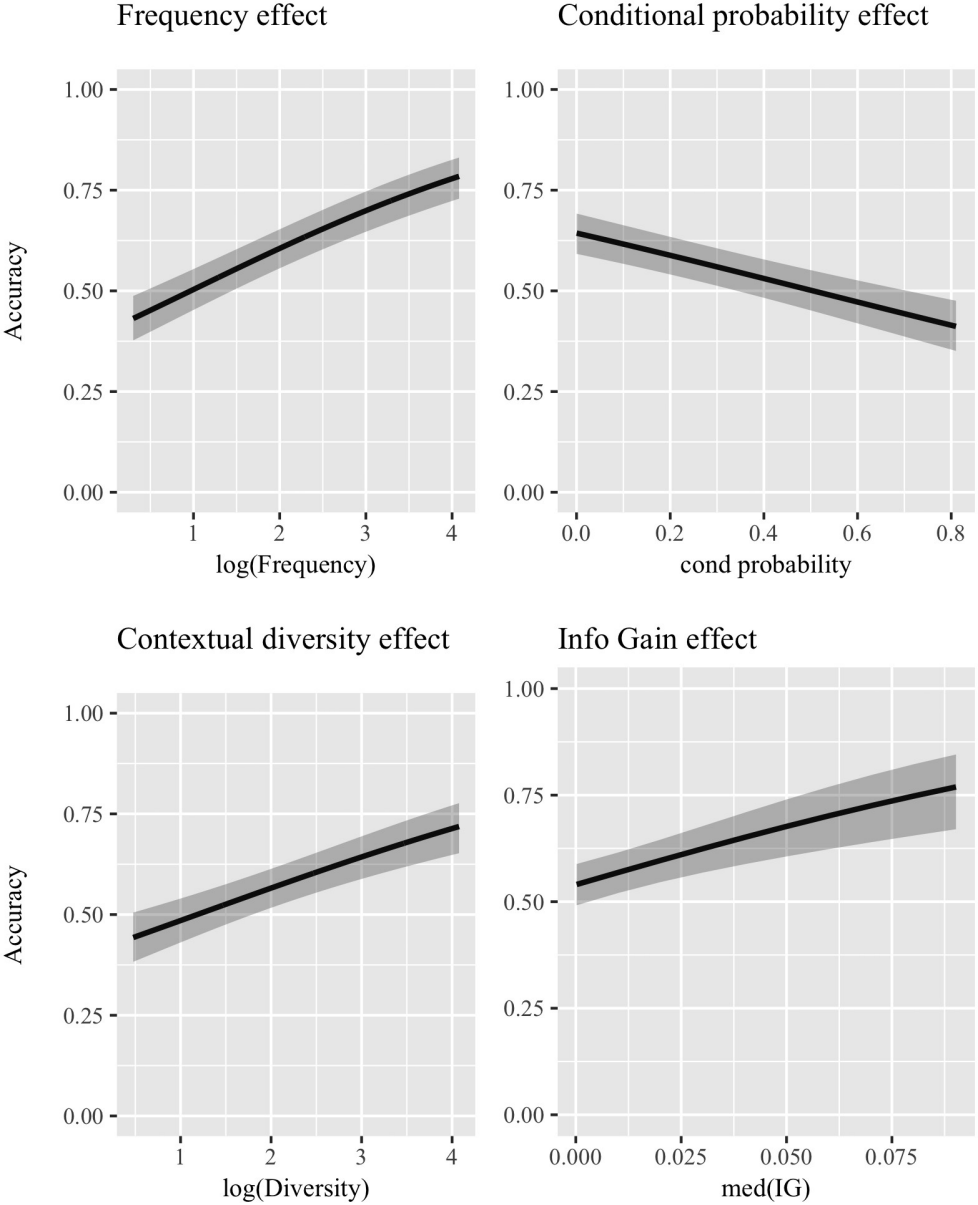
Logistic mixed-effect models for single predictors—Numeric overlap distance. Graphical representation of the effects highlighted by the logistic mixed-effect models which included *a single predictor* (inputted in the model together with Time, which is part of the baseline model) using categorization accuracy achieved with **numeric overlap** as distance metric as the dependent variable. Subplots are ordered according to the reduction in AIC caused by adding the predictor to the baseline model: the most important predictor is in the top-left panel, then top-right, bottom-left, and finally bottom-right. 95% confidence bands are shown.

Table 3 shows results for the individual analyses conducted using the categorization outcome based on numeric overlap as the dependent variable. The baseline model is the same as in Studies 1 and 2, with the random component including by-corpus random intercepts and by-word random intercepts and slopes. This entails that time is a fixed effect in the baseline model. Table 3 mirrors Table 1, providing *β* coefficients (*β*), standard errors (*se*), Z statistic (*Z*), AIC value, and marginal and conditional *r*^2^ (m*r*^2^ and c*r*^2^ respectively) for each predictor of interest. Fig 4 graphically presents the effects reported in Table 3.

**Table 3:** Logistic mixed-effect models for single predictors—Numeric overlap distance.

Predictor	*β*	*se*	*Z*	AIC	m*r*^2^	c*r*^2^
Baseline				43144	0.0006	0.5882
Frequency	0.4146	0.0452	9.173 (***)	43063	0.0095	0.6017
Conditional probability	-1.1684	0.1794	-6.512 (***)	43104	0.0037	0.5770
Contextual diversity	0.3240	0.0552	5.867 (***)	43112	0.0043	0.5977
Median IG	11.5745	2.7601	4.193 (***)	43130	0.0012	0.5870

Outcome of the logistic mixed-effect models fitted using accuracy obtained using **cosine** as the distance metric to identify nearest neighbors as the dependent variable. Each model includes *a single predictor* (together with Time, which is part of the baseline model). Significance codes:

(***): *p* < .001.

First, all predictors are significant at *p* < .001. The most important one appears to be frequency (*β* = 0.4146, *se* = 0.1169, *Z* = 9.173), with a positive effect. The second most informative predictor is conditional probability, with a negative effect (*β* = −1.1684, *se* = 0.1794, *Z* = −6.512). The following predictor is contextual diversity, with a positive effect (*β* = 0.3240, *se* = 0.0552, *Z* = 5.867). Finally, median IG also has a positive effect (*β* = 11.5747, *se* = 2.7601, *Z* = 4.193). The usual caveat about the magnitude of the coefficients of conditional probability and median IG applies, since their values exist on a smaller interval than the reference unit. In general, these results mirror the ones shown in Study 1, with the only difference that here frequency is the most important predictor, whereas diversity explains more variance when cosine is used. It can be observed, however, how both frequency and diversity have a positive effect using both distance metrics, while conditional probability has a negative effect. The effect of median IG confirms the assumption that *easy* words tend to co-occur with useful contexts.

**Fig 5:**
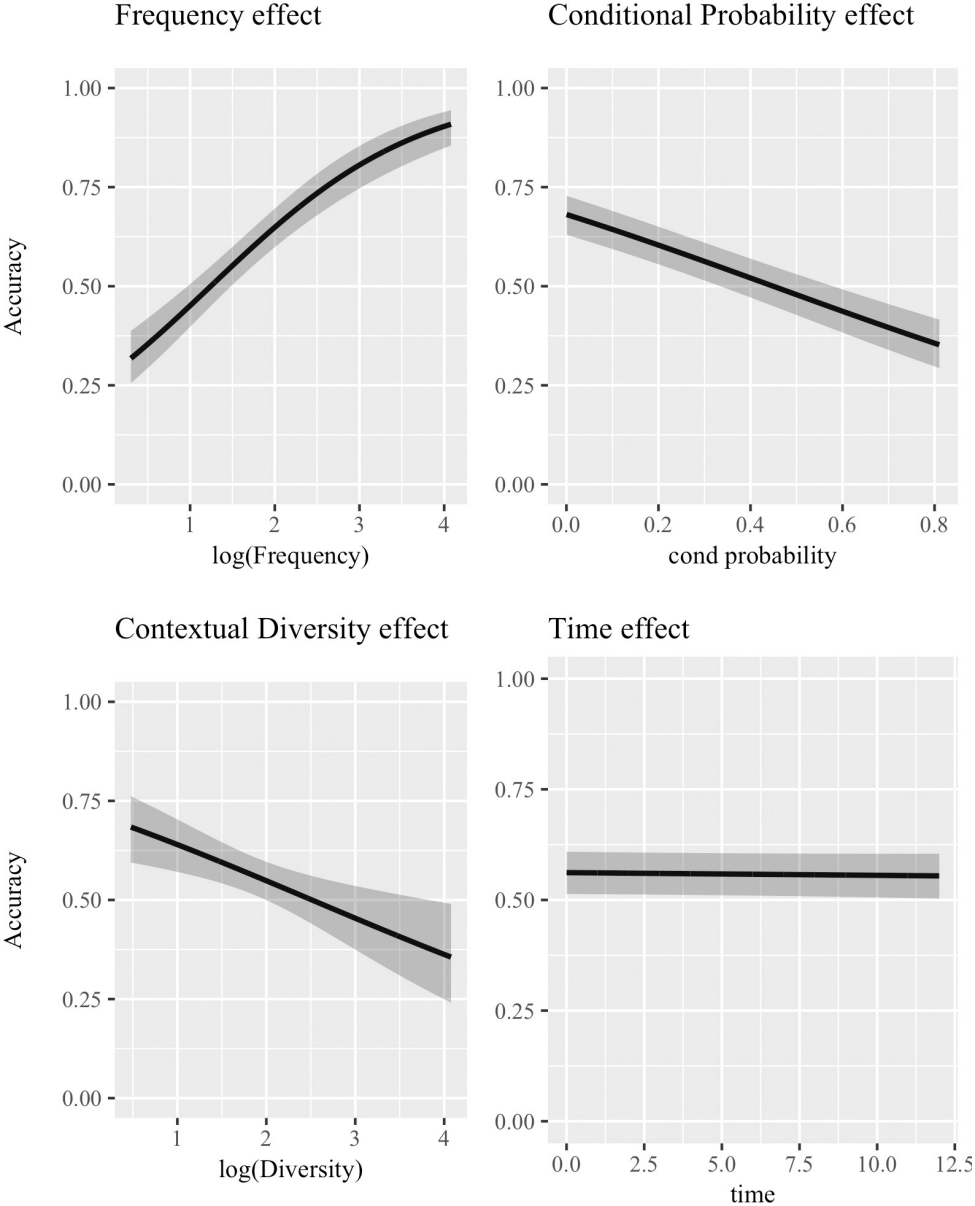
Logistic mixed-effect model including all predictors—Numeric overlap distance. Graphical representation of the effects arising from the logistic mixed-effect mode fitted considering *all significant predictors* at once and accuracy achieved using **numeric overlap** to retrieve nearest neighbors as the dependent variable. 95% confidence bands are shown for each effect, and predictors are ordered according to how much they improve the model fit (first top-left, then top-right, bottom-left, and bottom-right.

Table 4 provides results for the statistical analysis conducted using categorization achieved with numeric overlap as the dependent variable and including all significant predictors in the same statistical model, paralleling what was reported in Study 2 using cosine as the distance metric. The analysis was conducted as detailed for Study 2. The baseline model is the same as the one used for the previous analysis in Study 3. Fig 5 provides the graphical representation of the effects reported in Table 4. The model has a marginal *r*^2^ of 0.0132 and a conditional *r*^2^ of 0.5835, in line with what was observed for cosine-based categorization.

**Table 4:** Logistic mixed-effect model considering all predictors—Numeric overlap distance.

Predictor	*β*	*se*	*Z*
(Intercept)	0.2532	0.1494	1.695 (ns)
Time	-0.0050	0.0084	-0.595 (ns)
Frequency	0.8100	0.0975	8.311 (***)
Conditional Probability	-1.6879	0.1913	-8.821 (***)
Contextual diversity	-0.3793	0.1213	-3.127 (**)

Outcome of the logistic mixed-effect model fitted considering *all significant predictor*s at once on categorization outcomes obtained using **numeric overlap** as the distance metric to retrieve nearest neighbors. Significance codes:

(***): *p* < .001;

(**): *p* < .01;

(ns): *p* > .05.

First of all, even though it is required because of the necessity of by-word random slopes, time does not have a significant effect on categorization accuracy (*β* = −0.0050, *se* = 0.0084, *Z* = −0.595, *p* > .05). Turning to the other predictors of interest, Frequency has a significantly positive effect (*β* = 0.81, *se* = 0.0975, *Z* = 8.311, *p* < .001). On the contrary, conditional probability negatively and significantly affects categorization accuracy (*β* = −1.6879, *se* = 0.1913, *Z* = −8.821, *p* < .001). The least important predictor to still be significant is contextual diversity which has a negative effect once frequency and conditional probability are controlled for (*β* = −0.3793, *se* = 0.1213, *Z* = −3.127, *p* < .01).

### 5.2 Discussion

The individual analyses on numeric overlap-based categorization confirm the negative effect of conditional probability, suggesting that this is an underlying principle of word categorization. This effect remains negative and significant also in the full model, where frequency effects are controlled for. Moreover, median IG still has a positive effect, suggesting once more that it is indeed the case that *easy* words tend to co-occur with more useful contexts, regardless of the choice of distance metric. Finally, the amount of variance explained even by the full model stays remarkably low, confirming that, while significant, the predictors of interest only scratch the surface in explaining what makes words easier to categorize.

Importantly, when numeric overlap is used, frequency is the most important predictor, with a positive effect both in the individual and full model. The pattern is less clear cut when looking at diversity, which has a positive effect by itself, but negatively influence categorization accuracy once frequency is controlled for. Its positive effect in isolation matches the prediction formulated in [51] while the change in the direction of the effect once frequency is controlled for suggests the influence of collinearity and calls for further analyses.

**Fig 6:**
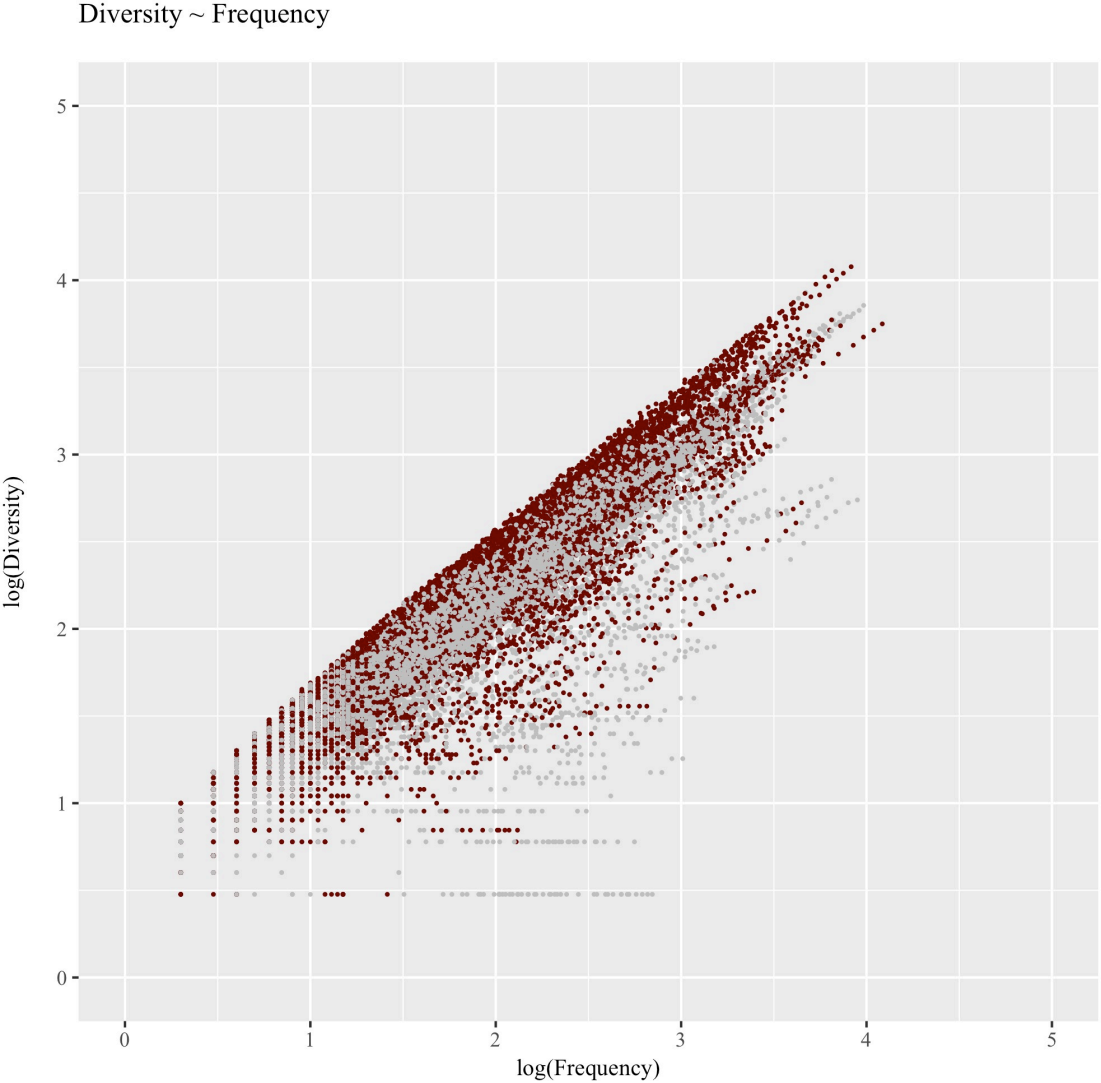
Relation between lexical diversity and frequency, with numeric overlap-based classification. Relation between Frequency and Diversity across words used in the categorization experiment analyzed in Studies 1, 2, and 3. Accuracy obtained using **numeric overlap** as the distance metric to compute nearest neighbors is shown highlighting misses in red.

To further analyze the relation between frequency and diversity a scatter plot was created, with frequency on the *x* axis, diversity on the *y* axis, and the categorization accuracy of each word shown using color (gray for correctly categorized words, red to highlight misclassified items, Fig 6). As is immediately evident, there is a solid red cloud on top, indicating that all the most diverse words were misclassified, regardless of their frequency. This categorization pattern, however, seems to affect the robustness of numeric overlap as a distance metric for the problem at hand rather than disqualify the effects reported in the current study regarding which distributional factors influence word categorization. Together with the lower categorization accuracy and the non-significant effect of time, the fact that the most diverse words are invariably misclassified suggests that numeric overlap is not suited to handle noisy vectors in an unsupervised and local *k*NN approach.

**Fig 7:**
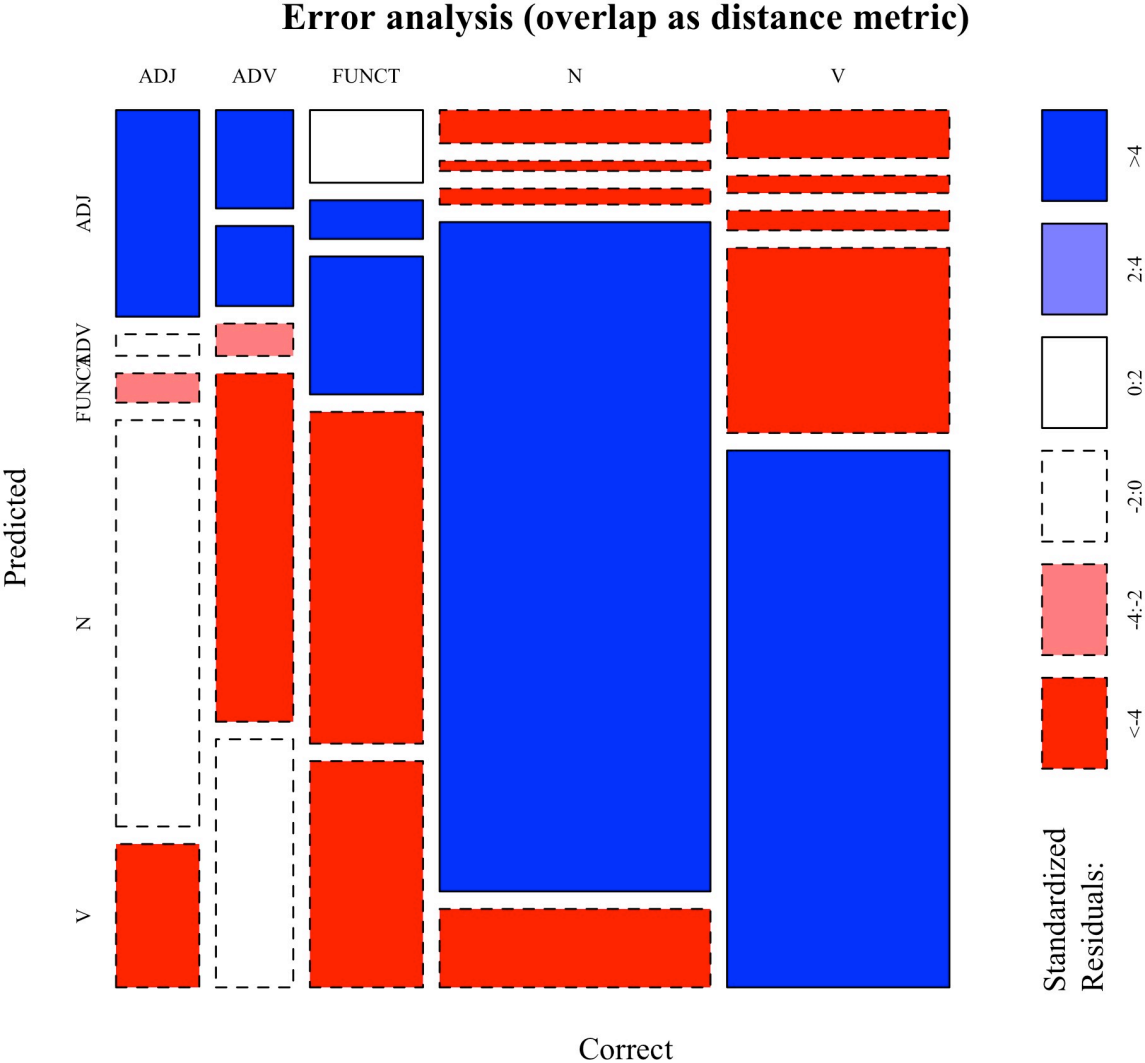
Numeric overlap-based classification—Error analysis. Mosaic plot showing the error analysis of the categorization experiment performed using **numeric overlap** as the distance metric to retrieve nearest neighbors. Blue cells indicate cases in which the observed frequencies significantly exceed the expected frequencies under the assumption of independence between Correct and Predicted PoS tags, while red mark the opposite scenario. ADJ: adjectives; ADV: adverbs; FUNCT: function words; N: nouns; V: verbs. For a detailed explanation of how to interpret the plot, refer to the main text.

This is confirmed by the error analysis provided in Fig 7, showing how categorization accuracy decreases especially for function words. Again, the cells on the main diagonal are all blue, showing that the correct predictions happen more often than it would be expected by chance. However, there are several more frequent errors as compared to the analysis provided for categorization using cosine. Many verbs are mistaken for nouns, even more adjectives are mistaken for nouns, function words are mostly mistaken for nouns and verbs. These patterns suggest that using numeric overlap as a distance metric did not lead to a partition of the word space which robustly and meaningfully reflects lexical categories.

However, the fact that categorization accuracy mostly changes for a specific category, i.e. function words, between the simulations conducted using cosine and those based on numeric overlap is worth a more thorough analysis, for two main reasons. First, it is observed that the relative importance of frequency and diversity changes with the distance metric. The error analysis suggests that categorization mostly changes for function words, which are highly diverse. Fig 6 highlights how the negative effect of diversity once frequency is controlled for arises because, at comparable frequencies, more diverse words are systematically misclassified. Second, it can be considered strange that function words are categorized so well using cosine, since children have a harder time creating categories for these words (see the study by [20] on determiners). Since the analyses presented so far considered function words as one big group, collapsing differences between several distinct categories, the high accuracy on function words is not taken to be representative of developmental patterns, but it is possible that it biased the observed effects. To make sure that any reported effect did not depend on having created an artificial category collapsing many linguistically relevant distinctions, the analyses in Studies 1, 2, and 3 were replicated after having removed all function words from the data set.

Results for this further replication are provided in Tables 5 and 6. The individual analyses, both with numeric overlap and cosine, do not change much, suggesting that when collinearity is left out of the picture, the effects reported throughout the paper are robust and informative, regardless of which lexical categories are considered and of which distance metric is chosen. The amount of explained variance as estimated using marginal and conditional *r*^2^ is also consistent with previous analyses. However, when all predictors were inputted in the same model, cosine and numeric overlap give rise to different patterns. In the cosine analysis, the picture does not change even after excluding function words: diversity keeps explaining more variance than frequency, which is no longer significant once diversity and conditional probability are controlled for. When analyzing numeric overlap-based categorization, on the contrary, results do change between the analysis on all words and the one which excludes function words. When focusing on content words, results become similar to those obtained with cosine: diversity has a stronger and more reliable effect than frequency, although the difference is subtle. The negative effect of conditional probability was observed in all analyses. Moreover, the effect of time is only significant in the cosine-based categorization, suggesting once again that numeric overlap is a poor choice for this task and that results obtained with it are less informative with respect to categorization patterns and what distributional factors affect them. In general, this further replication shows that the main difference between numeric overlap and cosine involves the categorization of function words, which correspond to the noisiest vectors. Once these vectors are left out, patterns become more similar, although the sensitivity of frequency and diversity effects to theoretically irrelevant experimental manipulations makes it harder to draw firm conclusions about how they relate to each other.

**Table 5:** Logistic mixed-effect models for single predictors (excluding function words).

Predictor	*β*	*se*	AIC
**Cosine**			
Baseline			29695
Contextual diversity	0.574	0.065	29619
Conditional probability	-1.521	0.196	29637
Frequency	0.377	0.053	29647
*Median IG*	3.650	4.111	29696
**Numeric overlap**			
Baseline			38384
Frequency	0.460	0.048	38294
Contextual diversity	0.565	0.060	38298
Conditional probability	-1.113	0.189	38352
Median IG	8.526	3.926	38379

*Individual effects* of the significant distributional predictors of interest for **cosine**-based and **numeric overlap**-based categorization after excluding all function words from the data set. Predictors are ordered according to the reduction in AIC over the baseline model and non-significant predictors are italicized.

**Table 6:** Logistic mixed-effect models including all predictors (escluding function words).

Predictor	*β*	*se*	Z
**Cosine**			
(Intercept)	1.089	0.163	6.697
Time	0.052	0.013	4.030
Contextual diversity	0.777	0.068	11.352
Conditional probability	-2.156	0.204	-10.561
**Numeric overlap**			
*(Intercept)*	0.005	0.168	0.031
*Time*	-0.004	0.009	-0.494
Frequency	0.264	0.124	2.125
Conditional probability	-1.879	0.201	-9.372
Contextual diversity	0.451	0.157	2.864

Effects of the *significant distributional predictors* of interest for **cosine**-based and **numeric overlap**-based categorization after excluding all function words from the data set when inputted in the same statistical model. Predictors are ordered according to the reduction in AIC brought over the simpler model. Predictors reported in italic are not significant, while all the others are significant at *p* < .05.

In conclusion, Study 3 provided evidence that changing the distance metric does indeed change results as far as the effects of frequency and contextual diversity are concerned. Diversity came out as the most important predictor when cosine is used and has a positive effect which completely accounts for the positive effect of frequency once both predictors are included in the same model. On the contrary, frequency explains more variance than diversity in numeric overlap-based categorization, and the effect of the latter predictor changes direction from positive to negative once frequency is controlled for. However, it was also observed that numeric overlap comes out as a worse choice to model categorization of words into lexical categories since it does not benefit from extra information provided by a larger corpus (the effect of time on categorization is not significant). Moreover, numeric overlap-based categorization was particularly derailed by function words which are very diverse vectors: this determined the change in direction of the effect once frequency was taken care of. After having excluded function words, the effects of frequency and diversity across the three experiments re-aligned: they are both positive in isolation and diversity explains more variance than frequency. The non-significant effect of time on numeric overlap-based categorization, though, remains, suggesting that measuring distributional similarity using numeric overlap does not allow the model to leverage the added information provided by more utterances, which makes it a poorer choice to model lexical category acquisition and study what drives it.

## 6 General discussion

This paper set out to answer the following research question: what distributional properties make words easier to categorize into lexical categories, such as nouns or verbs? In order to address this question, corpus-based computational simulations were carried out, using individual corpora of transcribed child-directed speech and a longitudinal approach. Mixed-Effect logistic regression analysis was used to model categorization accuracy as a function of three main distributional predictors: frequency, contextual diversity, and predictability. These were chosen to evaluate the impact of how often a word occurs, how many different distributional contexts it co-occurs with, and how predictable it is given co-occurring contexts. Results show that predictability has a robust negative effect, and that both frequency and diversity have a positive effect when considered in isolation, but their role and relative importance change when both are included in the same model, depending on how categorization is implemented and which target categories are considered.

The most interesting result concerns the negative effect of predictability, which matches results and predictions from previous studies. First, behavioral evidence presented by Matthews and Bannard [59] showed that children find it easier to categorize words when they co-occur with diverse contexts. This entails that more easily categorized words are harder to predict given the contexts they co-occur with. Second, the negative effect of conditional probability was predicted based on the observation that useful contexts tend to be lexically diverse, i.e. to co-occur with many different words [51]. This predicts the negative effect of predictability observed in the current work, since if a useful context is diverse, the individual words co-occurring with it are harder to predict.

The negative effect of predictability across all studies in this work seems to be an inherent property of categorization. Its effect is not affected by other predictors, by differences in the set of target categories, or in the distance metric used in the *k*NN categorization algorithm. This negative effect also makes intuitive sense: categories are more easily formed when items can be substituted for one another without violating strong expectations. It suggests that categorization is easier in the face of uncertainty: whenever distributional information is highly predictive of specific linguistic items, it is harder to see an opportunity to form a productive category. Moreover, this study confirms that the role of predictability in language learning extends beyond word segmentation and chunking where it was previously explored [52–58].

However, the negative effect of predictability on categorization accuracy is at odds with evidence from studies in sentence processing that report that more predictable words given the distributional contexts are processed more easily [83, 84]. As was previously discussed, there seem to be a tension between prediction and categorization such that what facilitates the former hampers the latter and vice versa. However, both are part of the larger problem of how language is efficiently processed, since prediction involves several levels, from specific items to categories thereof. One possibility is that low predictability combinations of distributional contexts and individual words force learners to group those words into larger clusters. Members of such clusters can be substituted with one another without violating expectations the closer they are in the distributional space. In this way, processing can later speed up when no item can be precisely predicted given a context because an array of legitimate items can. On the contrary, predictable combinations in development prevent the generalization and the formation of clusters of replaceable elements. Since items can be easily predicted, there is no need to reduce the uncertainty by grouping elements in larger clusters.

The effects uncovered for frequency and diversity are less clear, with mixed patterns observed depending on the distance metric used to retrieved nearest neighbors. In detail, when frequency and diversity are considered alone, their individual effects are both positive and significant, regardless of the chosen distance metric. This confirms the prediction formulated in [51]. However, when all predictors are entered at once in the same statistical model, differences emerge due to the choice of distance metric. When cosine is used, frequency does not explain any more variance than diversity; with numeric overlap (a more frequency sensitive distance metric than cosine), on the contrary, frequency explains more variance than diversity, whose effect becomes negative once frequency is controlled for. However, it was also observed that the biggest discrepancy between cosine-based and numeric overlap-based categorization concerns function words, which are in general very frequent and correspond to highly contextually diverse and noisy co-occurrence vectors. Since the category of function words was created as a bucket to keep the focus on content words, it conflated many linguistically different sub-categories, making the task of categorizing them considerably easier than the task faced by children. Given that this simplification might have biased results, the analyses from Studies 1, 2, and 3 were replicated by excluding all function words from the data set. The effects highlighted in Studies 1 and 2, modeling cosine-based categorization, were replicated even after removing function words. When numeric overlap-based categorization was analyzed, on the contrary, different patterns than those reported in Study 3 were observed. Diversity results as the most important predictor, in line with the analysis on cosine-based categorization, confirming that numeric overlap-based categorization was derailed by noisy vectors. The differences observed when considering or excluding function words suggest that they have a particular status when distributional information is considered, in line with evidence from Roy et al. [42] about word learning. This status should be investigated further, to better characterize distributional effects in lexical category acquisition.

All in all, it is hard to determine which of frequency and diversity has a stronger effect, since their influence on categorization accuracy depends on several factors, including (i) how the simulations are designed and implemented, (ii) which categories are used as targets and (iii) how such categories are defined. However, the reported analyses invariably show that diversity has a role next to, and often beyond, frequency. In line with several studies on different aspects of language acquisition [45–50, 59], this study shows that frequency does not suffice to account for which words are categorized better. Studies that only rely on frequency to decide which words to consider are likely to overlook important aspects of the problem, which were brought up by the reported analyses.

Finally, the positive effect of diversity fits with the negative effect of conditional probability in sketching categorization as a process that thrives on uncertainty. As was discussed, words can be more easily categorized when they are hard to predict given those contexts. The positive effect of diversity adds to this that categorization is also facilitated when a word co-occurs with many different distributional contexts. This implies that categorization is more effective (i) when learners can collect several pieces of evidence about the co-occurrence restrictions of words (since they co-occur with more contexts) and (ii) when no strong expectations are violated when substituting a word with another in a given context. As a matter of fact, categorization is a productive process that can only happen in the presence of variation: two words can form a category only when they share some property and can be replaced with one another.

This conclusion fits with evidence about word learning, where diversity and predictability were also shown to play a role [45, 47, 48]. Experiencing a word in a variety of different contexts was reported to help to recognize the word, both in terms of accuracy and speed [85, 86], suggesting an effect of semantic richness (similar to the effect of diversity in terms of neural correlates, as found by Vergara-Martinez et al [43]). However, Roy et al [42] found that words were acquired earlier when their context of occurrence was rather predictable, considering space, time, and linguistic contexts. Therefore, a word was learned sooner if its occurrences were concentrated at a certain time of the day (e.g. breakfast time), or in a certain part of the house (such as the kitchen), or across similar discourse contexts (words coherently referring to food and eating). Moreover, Johns et al [86] also found that repetitions in the same contexts induced more stable semantic representations. Therefore, it seems to be the case that frequency and diversity help word learning, and possibly lexical category acquisition, in different ways. Many, repeated occurrences of a certain item in the same context strengthen the impressions that the context is a defining property of the item, helping to predict the item from the context (and vice versa). On the contrary, the occurrence of an item in a variety of contexts blurs the boundaries of the item itself, since some co-occurrences could be spurious or uninformative, but helps recognizing and categorizing the item as a member of a broader category, which helps its recognition by activating the other, similar items.

The second important point brought up by this study is a theoretical one, having to do with how lexical categories were conceived throughout the study. In line with the proposal by Ambridge [60] that lexical categories are an illusory construct, the learning algorithm adopted in the reported simulation does not require lexical categories to be posited as independent entities with psychological validity to show categorical behavior [21]. The only requirement is a mechanism that computes and evaluates similarity across words, encoded as vectors of co-occurrences over distributional contexts. Words are not assigned to categories as in the study by Mintz [16], neither are categories assigned to words, as in the work by St. Clair et al [22]. Moreover, categories are not necessary for learning. In the framework adopted for the current studies, categorization only relies on similarity and proximity [87–89]. The current simulations show that using a completely unsupervised and local *k*NN approach, the space is partitioned in local regions that robustly reflect lexical categories, especially for nouns and verbs. Thus, learners could see an opportunity to use the word *porcupine* in the context *your*_X because the word *cat*, to which porcupine is observed to be distributionally very similar, co-occurred with it. At the same time, learners would not see the opportunity to use the word *engage* in the context *your*_X because it is found to be distributionally different from *cat*. This behavior would appear to be categorically constrained, but it does not require that the words *cat* and *porcupine* are assigned to the same category while *cat* and *engage* are not.

However, the decision to evaluate categorization using a set of gold-standard lexical categories may seem at odds with the idea that categories do not need to exist as independent entities for categorical behaviors to be observed. Nonetheless, a gold-standard evaluation provides a clear, easy to grasp picture of how the space defined by distributional co-occurrences reflects linguistic differences. Consequently, it is possible to evaluate which properties of the input influence successful categorization. If categorization is effective under a gold-standard evaluation, it means that the space encodes linguistic differences across words. However, it is necessary to extend this work by investigating whether distributional similarities which have been found to support the coarse grouping of words into lexical categories also capture more fine-grained distinctions. This could be done, for example, by using behavioral or imaging item-level measures of categorization which are at present not available to the best of our knowledge. It would be interesting, for example, to correlate the distributional similarity between two words with the degree to which children find it acceptable that the two words are substituted with one another, or with how surprised they are when finding a certain word in a novel context where the other word was observed. The reported evidence constitutes a first effort in analyzing categorization at the item level, in the hope that it spawns more efforts in this direction to better characterize what information supports lexical category acquisition.

Importantly, the observation that robust categorization arises from distributional information does not entail that lexical categories are acquired from distributional information only. Several studies have highlighted how other pieces of information contribute to the successful completion of the task, including phonology [90–92], semantics [93], and prosody [94, 95]. However, this work shows that it is possible to robustly infer lexical categories from distributional information using a completely unsupervised and local algorithm that does not need categories to be posited as independent constructs [60, 61].

### 6.1 Limitations and future work

A first limitation is to be found in the list of predictors investigated in this study, which is not meant to be exhaustive. On the contrary, frequency, diversity, and conditional probability were selected as a starting point for the main reason that they were found to significantly affect several phenomena in language acquisition, other than lexical category acquisition. It was therefore of interest to evaluate to what extent they also played a role in this area of language learning. However, many other distributional factors exist and should be investigated. This is made more pressing and necessary since the predictors considered in the current study did not explain much variance in categorization accuracy. Other distributional properties likely play a role, including what Roy et al. [42] term distinctiveness, i.e. the degree to which a certain word is consistently experienced in a specific context and not others. It was shown that this measure had a facilitatory effect on the age of first production of a word, with more distinctive words being produced earlier. This might interact with frequency, since function words tend to be experienced in a variety of different contexts, becoming less salient and potentially delaying the formation of categories for those words.

Moreover, the predictors considered here could be quantified differently. Frequency, for example, could be transformed into Zipf’s frequency [96], where the log transformation is applied to the standardized frequency per million words (the precise formula also involves the addition of a constant to ensure the interpretability of the scale (see the original study for details)) rather than to the absolute frequency count. However, in the context of a longitudinal study like the current one this transformation cancels the monotonically increasing nature of frequency, since a word which frequently occurs in the beginning of a corpus would have a higher Zipf frequency value at earlier time steps (when more input is processed, the frequency of the word would stay constant while the corpus size would get larger, reducing its standardized frequency). This way of operationalizing frequency would take relative frequency into account, and may be a fruitful way of expanding this work. However, it was chosen to operationalize frequency in the most straightforward way because of the exploratory nature of the study and the lack of precise, explicit hypotheses about its role in the context of lexical category acquisition. In a similar way, contextual diversity could encode the overlap between contexts, to better encode the amount of information that each new distributional context brings to the task [45, 85, 86]. Again, it was decided to adopt a simple approach because of the lack of previous research on the role of these predictors in the context of interest. Finally, operationalizing predictability as the average conditional probability of words given co-occurring contexts is not the only possibility. The median, maximum, minimum, or range of conditional probabilities for a word could all be investigated. Given that a negative effect was observed for the average, the minimum conditional probability of a word given co-occurring words seems more promising. After all, it may be the case that it is enough to encounter a word in just one context where the word is unpredictable to already have a substantial amount of information ot infer its category. Different ways of quantifying the predictors can and should be explored next to expanding the pool of predictors of interest, since it could offer more insights about the information children leverage to learn about lexical categories and how.

A further step should involve relaxing assumptions of linearity adopted for simplicity in the current analysis: this is especially true for the average conditional probability, since the Goldilocks effect reported in infants’ visual and auditory perception [97, 98] predicts that children will preferentially attend to stimuli that are neither too predictable nor too hard to predict, focusing on a sweet spot of predictability in between the two extremes. Too predictable information will be perceived as useless and boring, while too unpredictable information will be too hard to track. Extending this prediction to language learning and lexical category acquisition in particular could result in a non-linear effect of conditional probability and possible non-linear interactions with other predictors which could further explain which words are more easily categorized by children. Deploying more refined statistical techniques and considering non-linear effects will likely uncover more nuanced effects and possibly better explain lexical category acquisition in young children.

A final limitation involves the use of a single language and a single learning mechanism. First, evidence from different languages is required to test the generality of the reported effects beyond English, which was chosen as a target language due to the large availability of corpora and related analyses. Languages with richer morphology can provide different cues to categorization [39], which may then be influenced in other ways or by other factors. Second, the current analysis based on a count-based model that instantiates traditional Hebbian learning with no error feedback [99] should be expanded by also considering other classes of learning mechanisms, such as error-driven learning [100, 101], where the model does not simply store co-occurrences but actively tries to predict what will happen and adjust its beliefs accordingly [102–104]. It was shown how using two different distance metrics changed the observed effects: this makes an analysis that uses other learning mechanisms even more compelling and useful to assess the robustness of the reported effects. If the same results are found with different classes of learning mechanisms, it would be useful evidence to determine that an effect is robust and reliable. If, on the contrary, different results were found, it would provide useful evidence to determine which of the two algorithms provides the best predictions and reduce the space of possible explanatory models in the context of lexical category acquisition.

## 7 Conclusions

This study analyzed lexical category acquisition from distributional information, with the goal of determining which distributional properties make words easier or harder to categorize. Results show that categorization is easier when words are hard to predict given the contexts they co-occur with and tend to co-occur frequently and with a variety of different distributional contexts. This sketches categorization as a process that best occurs in the face of uncertainty: categories are formed when learners are not sure of which specific words will occur in a given context and can substitute several words for one another.
