# Correction: Lexical category acquisition is facilitated by uncertainty in distributional co-occurrences

**DOI:** 10.1371/journal.pone.0211594

**Published:** 2019-01-25

**Authors:** The PLOS ONE Staff

## Notice of republication

This article was republished on January 17, 2019 to remove a Supporting Information file that was incorrectly included in the originally published article. The publisher apologizes for the error. Please download this article again to view the correct version.
